# Ambient Temperature Effects on the Spring and Autumn Somatic Growth Trajectory Show Plasticity in the Photoneuroendocrine Response Pathway in the Tundra Vole

**DOI:** 10.1177/07487304231190156

**Published:** 2023-08-11

**Authors:** Mattis Jayme van Dalum, Laura van Rosmalen, Daniel Appenroth, Fernando Cazarez Marquez, Renzo T. M. Roodenrijs, Lauren de Wit, Roelof A. Hut, David G. Hazlerigg

**Affiliations:** * Arctic Seasonal Timekeeping Initiative, Department of Arctic and Marine Biology, UiT—the Arctic University of Norway, Tromsø, Norway; † Chronobiology Unit, Groningen Institute for Evolutionary Life Sciences, University of Groningen, Groningen, The Netherlands; ‡ The Salk Institute for Biological Studies, La Jolla, California

**Keywords:** seasonality, maternal photoperiodic programming, temperature, photoperiodic neuroendocrine system, pars tuberalis, hypothalamus, *Microtus oeconomus*, tundra vole, deiodinase, thyroid signaling, testosterone and somatic growth

## Abstract

Seasonal mammals register photoperiodic changes through the photoneuroendocrine system enabling them to time seasonal changes in growth, metabolism, and reproduction. To a varying extent, proximate environmental factors like ambient temperature (T_a_) modulate timing of seasonal changes in physiology, conferring adaptive flexibility. While the molecular photoneuroendocrine pathway governing the seasonal responses is well defined, the mechanistic integration of nonphotoperiodic modulatory cues is poorly understood. Here, we explored the interaction between T_a_ and photoperiod in tundra voles, *Microtus oeconomus*, a boreal species in which the main impact of photoperiod is on postnatal somatic growth. We demonstrate that postweaning growth potential depends on both gestational and postweaning patterns of photoperiodic exposure, with the highest growth potential seen in voles experiencing short (8 h) gestational and long (16 h) postweaning photoperiods—corresponding to a spring growth program. Modulation by T_a_ was asymmetric: low T_a_ (10 °C) enhanced the growth potential of voles gestated on short photoperiods independent of postweaning photoperiod exposure, whereas in voles gestated on long photoperiods, showing a lower autumn-programmed growth potential, the effect of T_a_ was highly dependent on postweaning photoperiod. Analysis of the primary molecular elements involved in the expression of a neuroendocrine response to photoperiod, thyrotropin beta subunit (*tshβ*) in the *pars tuberalis*, somatostatin (*srif*) in the arcuate nucleus, and type 2/3 deiodinase (*dio2*/*dio3*) in the mediobasal hypothalamus identified *dio2* as the most T_a_-sensitive gene across the study, showing increased expression at higher T_a_, while higher T_a_ reduced somatostatin expression. Contrastingly *dio3* and *tshβ* were largely insensitive to T_a_. Overall, these observations reveal a complex interplay between T_a_ and photoperiodic control of postnatal growth in *M. oeconomus*, and suggest that integration of T_a_ into the control of growth occurs downstream of the primary photoperiodic response cascade revealing potential adaptivity of small herbivores facing rising temperatures at high latitudes.

In species living in temperate and boreal zones, the scheduling of growth, development, and reproduction is contingent on the annual cycle of seasonal environmental change stemming from Earth’s orbit of the Sun. This has led to the evolution of seasonal synchronization mechanisms reliant on changes in the daily photoperiod as a synchronizer (Dardente et al., 2014; Hazlerigg and Simonneaux, 2015; Wood and Loudon, 2018). In addition, to a degree that varies between species, proximate factors such as nutritional status, ambient temperature (T_a_), predation, and social interactions modulate seasonal scheduling, giving phenotypic plasticity in the face of year-to-year variation in environmental seasonality (Bronson, 1985, 2009; Visser et al., 2010).

It has been argued based on life-history considerations that modulatory effects conferring plasticity are likely to be of increased importance in smaller short-lived species (e.g., nonhibernating rodents) than in larger species that survive and reproduce over multiple years (e.g., cervids) (Bronson, 1988). Accordingly, we have focused on microtine rodents as a suitable group in which to explore interactions between photoperiod and nonphotic influences on seasonal physiology.

Voles of the genus *Microtus*, the most speciose mammalian genus, inhabit large distribution ranges from the equator to the high arctic (Brunhoff et al., 2003; Jaarola et al., 2004), thereby meeting a variety of seasonal environments. In most *Microtus* species living at temperate and high latitudes, breeding starts in spring and ends in autumn with considerable variation in the onset and offset of the breeding season between species, populations, and years (Balčiauskas et al., 2012; Ergon, 2007; Gliwicz, 1996; Tast, 1966). High vole population densities delay the breeding onset and reduce the probability of maturation (Ergon et al., 2001), whereas a high predation risk advanced the onset of the breeding season (Gliwicz, 2007). Ambient temperature serves as an additional time cue in some birds (Visser et al., 2009), and it interacts with photoperiod on the GnRH system in prairie voles (Kriegsfeld et al., 2000). Voles breed in cohorts with spring-born pups growing and maturing fast to reach a high summer body mass and reproduce during the same breeding season. By contrast, pups born later in the season grow slower, maintain a lower body mass, overwinter in juvenile state, and sexually mature shortly before the onset of the next breeding season (Eccard and Herde, 2013; Gliwicz, 1996).

In the common vole (*Microtus arvalis*), we recently reported that photoperiodic experience in early life shapes postnatal reproductive development, preparing the voles for either rapid reproduction or overwintering. This developmental trajectory is determined by interactive effects of photoperiod exposure experienced in utero, via the maternal melatonin signal, and in the juvenile period, directly through the pup’s own photoneuroendocrine system (Horton and Stetson, 1992; van Rosmalen et al., 2021). Furthermore, we have demonstrated that temperature influences on this response are associated with changes in thyroid hormone deiodinase gene (*dio2* and *dio3*) expression in the basal hypothalamus (van Rosmalen et al., 2021)—establishing a point of intersection with the photoperiodic response one step removed from proximate actions of photoperiod on thyroid stimulating hormone (*tshβ*) expression in the *pars tuberalis* (PT) of the pituitary gland (Dardente et al., 2014).

We have also begun to investigate photoperiodic influences in a species with a more northerly paleogeography, the tundra vole (*Microtus oeconomus*) (Conroy and Cook, 2000). In contrast to the common vole, early life photoperiod has a smaller impact on postnatal reproductive development in *M. oeconomus*, while there are clear photoperiodic influences on somatic growth during the juvenile period, not seen in *M. arvalis* (van Rosmalen et al., 2020). These results suggest that photoperiod controls the somatic and gonadal axes differently between the two species.

The interaction of photoperiod and other environmental factors with gonadal development is better understood (for review, see Hazlerigg and Simonneaux, 2015) than with body mass and energy metabolism. The growth hormone (GH) axis is one potential photoperiod-controlled pathway. GH-releasing hormone (GHRH) from the arcuate nucleus stimulates the release of GH from the pituitary, but this is inhibited by somatostatin, also referred to as somatotropin release inhibiting factor (SRIF) (Dumbell et al., 2015). Somatostatin expression in the arcuate nucleus is strongly regulated by photoperiod in Siberian hamsters but the effects of other environmental factors such as ambient temperature remain to be investigated (Herwig et al., 2012; Petri et al., 2016).

In this study, our aim was 3-fold. First, we wished to extend our initial characterization of early life photoperiod influences on somatic growth in *M. oeconomus* to resolve between the effects of gestational and neonatal photoperiod. Second, by combined manipulation of photoperiod and ambient temperature, we sought to assess the extent to which photoperiodically programmed seasonal growth trajectories show plasticity. Finally, by analysis of gene expression in the basal hypothalamus and PT, we sought to determine the extent to which modulatory effects of temperature on juvenile growth reflect effects on the neuroendocrine machinery of the primary photoperiodic response.

## Materials and Methods

### Animals and Experimental Procedures

All experimental procedures were carried out according to the guidelines of the animal welfare body (IvD) of the University of Groningen conform to Directive 2010/63/EU and approved by the Centrale Commissie Dierproeven (CCD) of the Netherlands (CCD license number: AVD1050020171566). Tundra or root voles (*M. oeconomus*) were obtained from four different areas in the Netherlands (van de Zande et al., 2000). The population has been kept in the laboratory as an outbred colony at the University of Groningen, which provided all voles used in this study. Adult and weaned voles were individually housed in transparent plastic cages (15 × 40 × 24 cm) provided with sawdust, dried hay, an opaque PVC tube, and ad libitum water and food (standard rodent chow; Altromin #141005). The experiments were carried out in temperature-controlled chambers in which ambient temperature (T_a_) and photoperiod were manipulated as described below.

**Figure 1.**
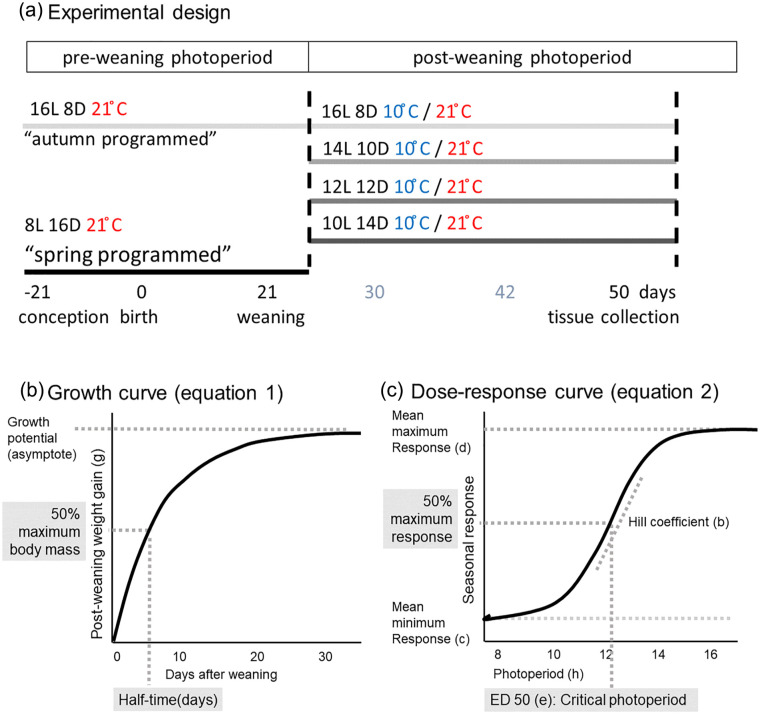
Experimental design and visualization of curve-fitting parameters. (a) Experimental design: conception, gestation, birth, and lactation took place under either 16 h of light (i.e., autumn-programmed) or 8 h of light (i.e., spring-programmed) at 21 °C. At the day of weaning (21 days old), voles from both preweaning photoperiods were transferred to four different postweaning photoperiods at either 10 °C or 21 °C resulting in eight different postweaning treatments. Tissue collections took place when voles were 50 days old. (b) Standard von Bertalanffy asymptotic growth function applied on the body mass data of each individual vole. (c) Four-parameter log-logistic function fitted to the mean seasonal response to photoperiod.

The voles used in the experiment (102 males) were gestated and born at 21 °C under either a short photoperiod (SP, 8 h of light/24 h: early breeding season, hereafter termed “spring-programmed”) or a long photoperiod (LP, 16 h of light/24 h: late breeding season, hereafter termed “autumn-programmed”) and weaned at an age of 21 days old. After weaning, voles were transferred to either 10 °C or 21 °C and a range of different photoperiods, a laboratory equivalent to different seasonal conditions (Figure 1a). Postweaning photoperiods (postPPs) were (hours light: hours dark): 16L:8D, 14L:10D, 12L:12D, 10L:14D. Hence, spring-programmed voles experienced a postweaning increase in photoperiod, while autumn-programmed voles experienced a postweaning decrease in photoperiod. All voles were weighed when 7, 15, 21, 30, 42, and 50 days old.

### Tissue Collection

At the age of 50 days, and ~17 h after lights-off, voles were sedated with CO_2_ and then decapitated. Trunk blood was collected directly from each individual into heparinized tubes. Blood samples were left on ice until centrifugation (10 min, 2600 × g, 4 °C). Plasma was transferred to a clean tube and stored at −80 °C until hormonal assay. Whole brains were carefully dissected to include the proximate pituitary stalk including the PT. Within 5 min after decapitation, brains were slowly frozen on a brass block surrounded by liquid N_2_. Brains were stored at −80 °C until proceeded to in situ hybridization. Reproductive organs were dissected, cleaned of fat, and wet masses of paired testes weight were measured (±0.0001 g).

### Radioactive In Situ Hybridization

A detailed description of the in situ hybridization protocol can be found elsewhere (Lomet et al., 2018). Briefly, 20-µm coronal brain sections were cut on a cryostat in caudal to rostral direction, starting from the mammillary bodies to the optic chiasm, to cover the area of the hypothalamus and third ventricle. Sections were mounted onto precoated Superfrost Plus slides (Thermo scientific: ref J1800AMNZ) with 6-10 sections per slide and 10 slides per individual. Antisense riboprobes of rat *tshβ* (GenBank accession no. M10902, nucleotide position 47-412), *M. arvalis dio2* (GenBank accession no. JF274709, position 1-775) and *M. arvalis dio3* (GenBank accession no. JF274710, position 47-412) were transcribed from linearized cDNA templates. Incorporation of 35^S^-UTP (Perkin Elmer, Boston, MA, USA) was done with T7 polymerase (*dio2* and *dio3*) and T3 polymerase (*tshβ*), resulting in 0.5-1.5 × 10^6^ counts per minute per microliter, calculated to have 10^6^ counts/min/slide. All slides were fixed in paraformaldehyde, acetylated, and hybridized with radioactive probes overnight at 56 °C.

Slides were washed in sodium citrate buffer the next day to remove nonspecific probe and then dehydrated in ethanol solutions, followed by air-drying. The slides were exposed to an autoradiographic film (Kodak, Rochester, NY, USA) for 9 days (*dio2* and *dio3*) or 11 days (*tshβ*) and developed with Carestream Kodak autoradiography GBX Developer/replenisher (P7042-1GA, Sigma) and fixer (P7167-1GA, Sigma). Films were scanned with an Epson Perfection V800 Photo scanner at 2400-dpi resolution along with a calibrated optical density strip (T2115C; Stouffer Graphic Arts Equipment Co., Mishawaka, IN, USA). Analysis of integrated optical density (IOD) was done with software ImageJ, version Fuji (NIH Image, Bethesda MD, USA). The section with the strongest signal was selected to represent each animal.

### Nonradioactive In Situ Hybridization

Nonradioactive in situ hybridization using digoxigenin-labeled riboprobes was performed to assess somatostatin (*srif*) expression in the arcuate nucleus of the mediobasal hypothalamus. Plasmids containing the rat somatostatin probe (135-465 of GenBank NM012659, 95% homology with *M. oeconomus* mRNA sequence, probe kindly provided by Dr. Paul Klosen) was used to transcribe the probe from PCR-produced templates in the presence of digoxigenin-labeled nucleotides (Roche, Meylan, France).

To avoid background differences in the staining, the whole set of slides was stained at once. The sections were postfixed with 4% formaldehyde, acetylated for 10 min in 100-mM triethanolamine and 0.25% acetic anhydride, and delipidated with 0.1% Triton X-100. The tissue was then hybridized with 200-ng/mL of labeled antisense probe in 50% formamide, 5× saline sodium citrate (SSC), 5× Denhardt’s solution, 250 µg/mL of baker’s yeast tRNA, and 200-µg/mL herring sperm DNA for 14 h at 60 °C. Sequential stringency washes were performed at 55 °C with 5× SSC (5 min), 2× SSC (1 min) and 0.2× SSC 50% formamide (30 min), and finally 0.2× SSC (5 min) at room temperature. The digoxigenin tag was detected using an alkaline phosphatase-coupled anti-digoxigenin antibody (1:3000; Roche). Alkaline phosphatase activity was visualized with a mixture of nitro blue tetrazolium/bromo-chloro-indolyl phosphate and stopped 3 h later before the staining intensity reached saturation. Hybridization with corresponding sense probes gave no signal, indicating specificity of the antisense probe.

Micrographs were acquired with a slide scanner (Olympus, VS120, Tokyo, Japan), using a 20× objective. All pictures were taken in one session with identical lighting conditions for all voles. First, the arcuate nucleus region was extracted using Qupath (version 0.4.1., Bankhead et al., 2017) and then ImageJ was used to determine the mean intensity of the in situ hybridization signal using a fixed squared size.

### Hormone Analysis

Plasma testosterone levels were measured in a mouse testosterone enzyme-linked immunosorbent assay according to manufacturer’s instructions (ADI-900-065; Enzo Life Sciences, New York, NY, USA). Sensitivity: 5.67 pg/ml, intra-assay coefficient of variation: 10.8%, interassay coefficient of variation: 9.3%.

### Fitting of Growth Curves

Postweaning growth in individual voles was modeled using a standard von Bertalanffy asymptotic growth function:

(1) $$W_{t}=W_{\infty }*\left(1-e^{-kt}\right),$$

where *W_t_* is the weight at time *t*, *W__∞__* is the growth potential (asymptote; the theoretical maximum size which an individual will attain), and *k* is the rate constant for reaching the asymptotic potential. The half-time was calculated as the time taken to attain 50% of the projected maximum weight. The day of weaning was set as *t* = 0 and mass at *t* = 0 was subtracted to give a zero baseline (Figure 1b). A summary of curve fits, giving *W_∞_* and half-times (ln[2])/*k*), can be found in Table S1.

### Calculation of Critical Photoperiod for Gene Expression Responses

Four-parameter log-logistic functions were fitted through the data to describe the response to photoperiod as a dose-response relationship.

(2) y=d+(c–d)1+(xe)^b.

In which *b* = slope parameter (hill coefficient), *c* = minimum, *d* = maximum, and *e* = 50% maximal response, where ED50 is defined as the inflection point of the curve. Critical photoperiod (CP) was estimated by the ED50 from fitted dose-response curves (Figure 1c). All fitted dose-response curve parameters can be found in Table S2.

### Analysis of Variance and Post Hoc Testing

The effects of postPP, T_a_, and interactions were determined within spring- and autumn-programmed experimental groups using two-way analysis of variance (ANOVA). To detect differences in the growth rate between groups, we used repeated-measures ANOVAs. The effect of somatostatin expression and T_a_ on growth and body mass was tested using a generalized linear model.

Data were log transformed for *dio2*, *dio3*, paired testes mass, and testosterone. Where appropriate, post hoc testing was performed using Tukey’s or Šidák’s test. Statistical significance was determined at *p* < 0.05 and results in the text are given as mean ± standard error of the mean (SEM). For all variables except somatostatin curve fitting, statistical analyses and figures were generated using GraphPad Prism v9. Somatostatin curve fitting, statistical analysis, and regression plots were done in R studio (RStudio Team, 2020) using the packages tidyverse (Wickham et al., 2019), drc (Ritz et al., 2015), and lsmeans (Lenth, 2016).

## Results

We have measured the postweaning growth and gene expression response to photoperiod and temperature in either spring- or autumn-programmed voles. The experimental design is summarized in Figure 1a.

### Somatic Growth

Photoperiodic conditions during gestation and prior to weaning (spring and autumn program) had no significant effect on body mass recorded at weaning (spring voles 16.5 ± 0.33 g; autumn voles 17.4 ± 0.49 g, *t*_73_ = 1.58, *p* = 0.12).

**Figure 2.**
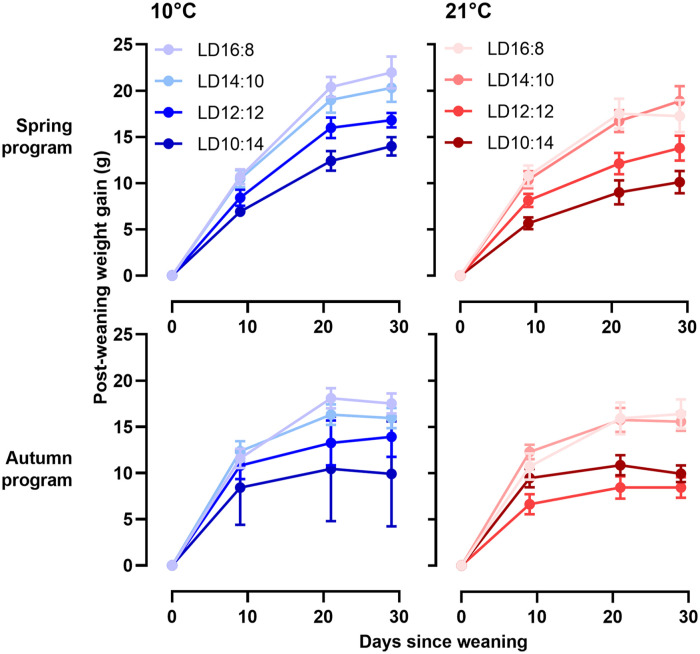
Postweaning growth curves in relation to photoperiod and ambient temperature. Postweaning growth curves for four different postweaning photoperiods at 10 °C (blue) or 21 °C (red) in both spring-programmed (LD8:16, top panel) and autumn-programmed (LD16:8, bottom panel) voles. Data are plotted as mean ± SEM (*n* = 4-8). Abbreviation: SEM = standard error of the mean.

**Figure 3.**
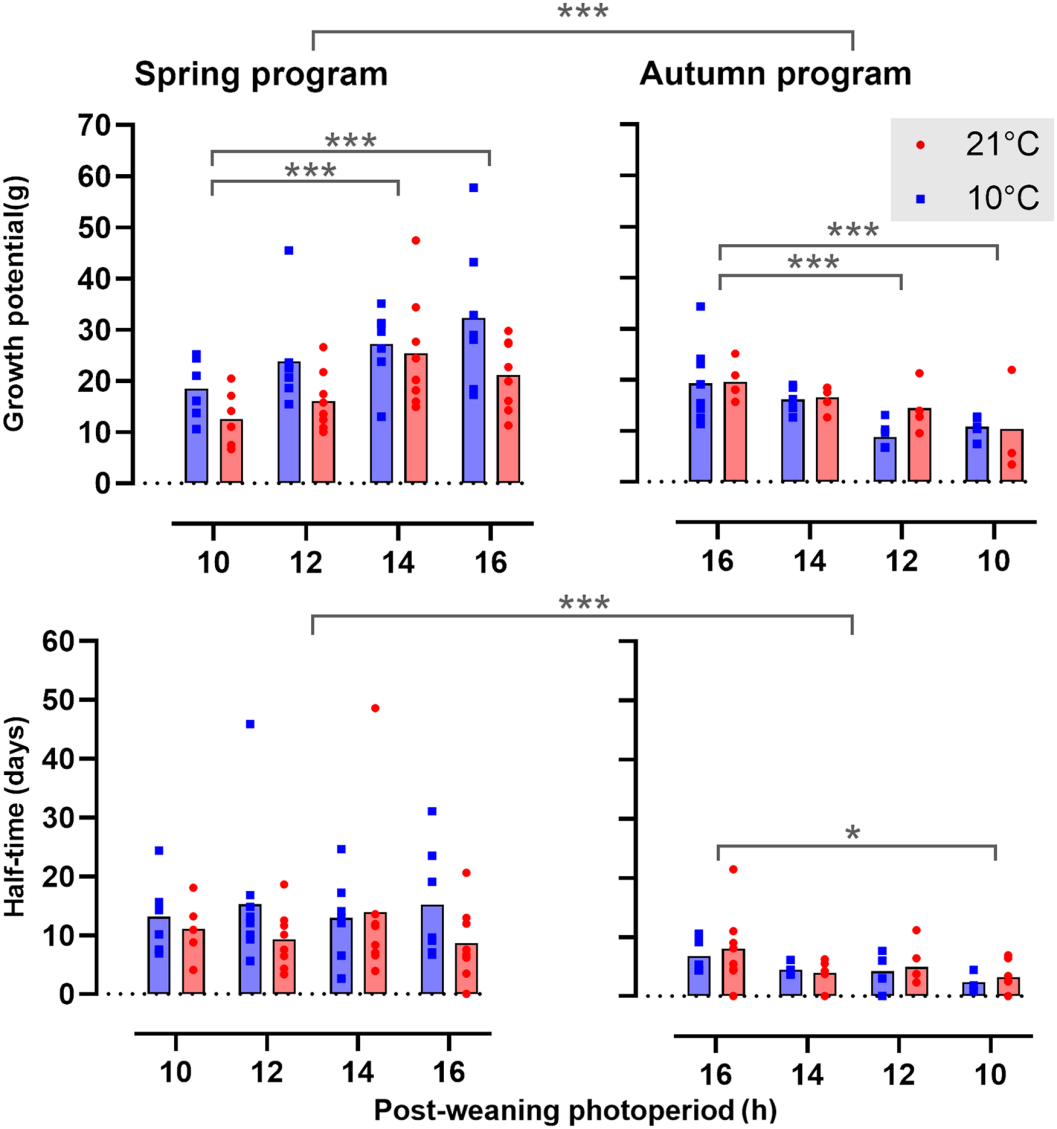
Mean growth potential and half-time in spring- and autumn-programmed voles. Growth potential is the asymptotic weight in the von Bertalanffy function, represented in the top panels, half-time (days) to reach the 50% maximum of the growth potential is shown in the lower panels for spring-programmed (left), and autumn-programmed (right) voles housed in postweaning ambient temperatures of either 10 °C (blue squares) or 21 °C (red circles). Significance levels are indicated: *p < 0.05. ***p < 0.001.

The effects of ambient temperature (T_a_) and postPP on postweaning somatic growth are summarized graphically in Figure 2, with curve fit parameters in Table S2. Overall, the growth potential, defined as the asymptotic weight in the von Bertalanffy function (Equation 1, Figure 1b), was consistently higher in spring-programmed voles (range: 7-58 g) than in autumn-programmed voles (range: 3-34 g) depending on postweaning conditions; postPP similarly had a strong influence on postweaning growth potential (spring, postPP: *F*_3,52_ = 5.37, *p* = 0.003; autumn, postPP: *F*_3,33_ = 7.46, *p* < 0.001) with the highest asymptotic weights on LD16:8 being some 1.5-fold greater than corresponding values under LD10:14, across all combinations of preweaning photoperiod (prePP) and ambient temperatures. In spring-programmed voles, across all postPPs (Figure 3), low T_a_ (10 °C) in the postweaning phase increased growth potential by up to approximately 40% compared with voles raised after weaning at 21 °C (T_a_: *F*_1,32_ = 9.21, *p* = 0.004, ns for T_a_ × postPP interaction). By contrast, T_a_ had no effect on growth potential in autumn-programmed voles (Figure 3).

Overall, postweaning half-times (in days, see Figure 1b) were significantly longer in spring (12.43 ± 1.15) than in autumn (5.04 ± 0.61) (prePP: *F*_1,97_ = 25.24, *p* < .0001 and ns for T_a_ and T_a_ × prePP), indicating slower geometric approach to maximum size in spring than in autumn. Only in autumn, short postPPs further reduced half-times (Figure 3, postPP: *F*_3,32_ = 2.92, *p* = 0.05, ns for T_a_ and T_a_ × postPP).

### Photoneuroendocrine Pathway Gene Expression

**Figure 4.**
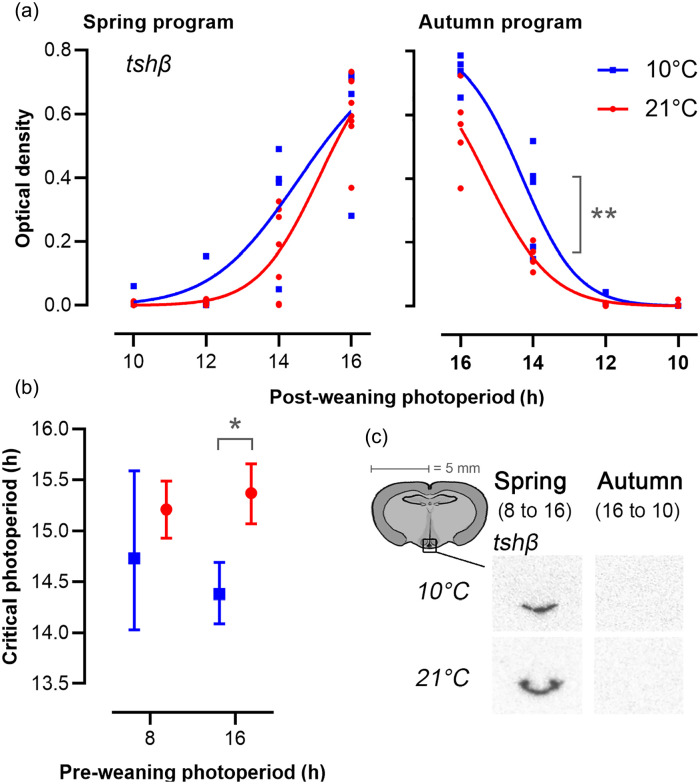
Expression of *tshβ* in the *pars tuberalis*. (a) Each data point represents optic density measurements from individual voles. Lines are best-fit curves for four-parameter log-logistic functions (Equation 2), as described in the methods. The left panels represent spring-programmed voles (gestated and raised to weaning under 8L) and the right panels represent autumn-programmed voles (gestated and raised to weaning under 16L) at 10 °C (blue squares) or 21 °C (red circles). All photoperiod treatments were significantly different from each other, except LD10:14 vs LD12:12 in both spring and autumn. (b) Critical photoperiod derived from fitted dose-response curves (Tables S1, S2) for *tshβ*; data are mean ± 95% CL (*n* = 4-8). (c) Representative images showing localization of mRNA by radioactive in situ hybridization for *tshβ* in the PT for the minimum and maximum expression levels during increasing (spring) and decreasing (autumn) photoperiods. Significance levels are indicated: *p < 0.05. **p < 0.01.

The expression of *tshβ* in the PT followed a sigmoid relationship to postPP (Figure 4, Table S2) from which CP for the primary photoperiodic response could be estimated. In spring-programmed voles, this represents the CP for induction of a long day increase in *tshβ* expression, while in autumn-programmed voles, this represents the CP for short day suppression of *tshβ* expression. In spring-programmed voles, CP was not significantly affected by T_a_ (10 °C CPspring = 14.73, 95% confidence interval [CI] = 14.01-15.46, *R*^2^ = 0.76; 21 °C CPspring = 15.21, 95% CI = 14.93-15.49, *R*^2^ = 0.88). Strong individual variation at LD14:10 at 10 °C led to a large spread around the estimated spring CP. In autumn-programmed voles, low T_a_ significantly decreased the CP for suppression of *tshβ* expression by about 1 h (10 °C CPautumn = 14.38, 95% CI = 14.09-14.96, *R*^2^ = 0.93; 21 °C CPautumn = 15.38, 95% CI = 15.12-15.3; *R*^2^ = 0.93 *p* < 0.05 for model comparison).

**Figure 5.**
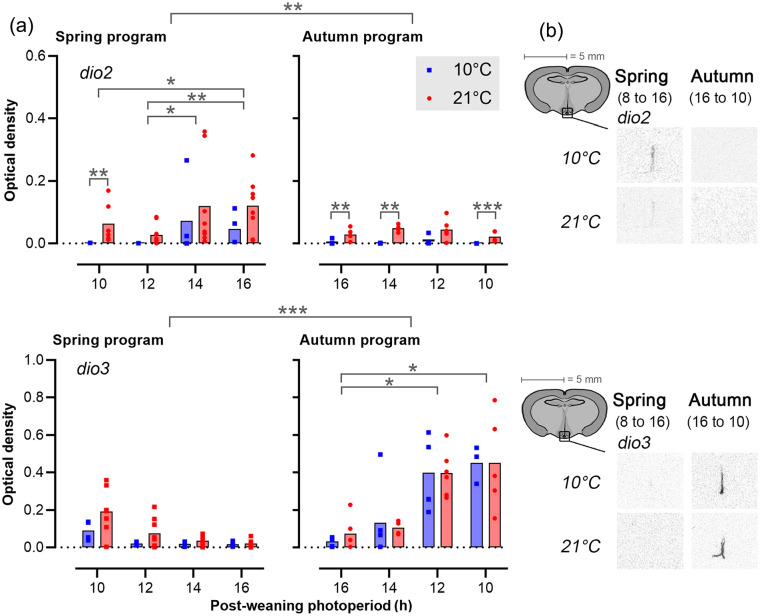
Expression of *dio2* and *dio 3* in the tanycytes. (a) Bar plots showing *dio2* and *dio3* expression in tanycytes (mean, dots are individuals). The left panels represent spring-programmed voles (gestated and raised to weaning under 8L) and the right panels represent autumn-programmed voles (gestated and raised to weaning under 16L) at 10 °C (blue squares) or 21 °C (red circles). (b) Representative images showing localization of mRNA in the median eminence region for the minimum and maximum expression levels during increasing (spring) and decreasing (autumn) photoperiods. Significant effects are indicated: *p < 0.05. **p < 0.01. ***p < 0.001.

**Figure 6.**
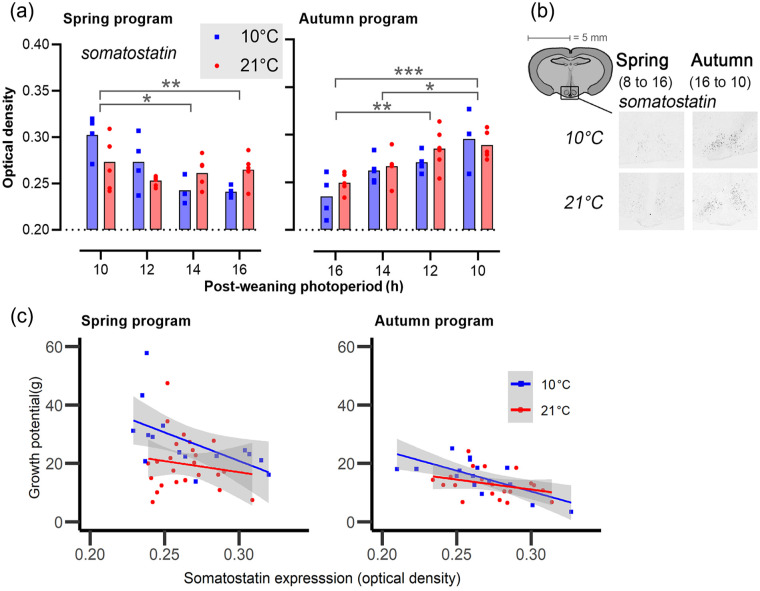
Expression of somatostatin (*srif*) in the arcuate nucleus. (a) Bar plots showing somatostatin expression in the arcuate nucleus. The left panels represent spring-programmed voles (gestated and raised to weaning under 8L) and the right panels represent autumn-programmed voles (gestated and raised to weaning under 16L) at 10 °C (blue squares) or 21 °C (red circles). (b) Representative images showing localization of mRNA by nonradioactive in situ hybridization in the arcuate nucleus. (c) Somatostatin (*srif*) expression (X-axis) against growth potential in g (Y-axis) for spring-programmed (left) and autumn-programmed (right) voles in which blue represents postweaning ambient temperatures of 10 °C and red at 21 °C. Gray areas indicate the 95% confidence levels of the regression line, and the data points are individual voles. Significant effects are indicated: *p < 0.05. ** *p* < 0.01. ***p < 0.001.

In contrast to *tshβ*, sigmoid model fits were poor descriptors of the patterns of *dio2* (*R*^2^ = 0.11-0.0), *dio3* (*R*^2^ = 0.47-0.60), and somatostatin (*R*^2^ = 0.01-0.02), gene expression across the postPP regimes (Figures 5 and 6, Table S2), but clear preweaning and postweaning effects were nonetheless observed. Overall *dio2* (Figure 5) expression was markedly higher in spring-programmed voles (0.06 ± 0.01) compared with autumn-programmed voles (0.02 ± 0.004), while the inverse was observed for *dio3* (spring: 0.06 ± 0.01, autumn: 0.24 ± 0.04).

Within spring-programmed voles, *dio2* was sensitive to postPP with the highest expression levels in LD16:8 voles being up to two orders of magnitude higher than levels in voles raised after weaning on LD10:14 (postPP: *F*_3,38_ = 6.06, *p* = 0.002). Across all postPPs, spring-programmed voles raised after weaning at a higher T_a_ generally had higher *dio2* expression than their low T_a_ counterparts (T_a_: *F*_1,38_ = 26.17, *p* < 0.0001). Although there was no significant postPP × T_a_ interaction under two-way ANOVA, the effect of T_a_ on spring program *dio2* expression was most apparent at short postPP, where expression appeared clamped at basal / background levels at 10 °C. In autumn-programmed voles, *dio2* expression was uniformly low across all postPPs, but a positive effect of increased T_a_ could still be observed (T_a_: *F*_1,29_ = 30.42, *p* < 0.0001), and as in the short postPP spring program voles, this appeared to be due to a clamping down of *dio2* expression to background levels in 10 °C voles.

In contrast to *dio2, dio3* (Figure 5) appeared to be highly sensitive to postPP (spring: *F*_3,38_ = 3.91, *p* = 0.02, autumn: *F*_3,29_ = 9.30, *p* < 0.001) but insensitive to T_a_. Within the spring-programmed voles, *dio3* was detectable in LD10:14 and 12:12, but suppressed to background levels on the two longer postPPs. In autumn-programmed voles, *dio3* was detectable at longer photoperiods than in spring, with several individuals showing above baseline expression on LD14:10 and the following shorter photoperiods.

### Somatostatin (Srif) Expression in the Arcuate Nucleus

Like with *dio2* and *dio3*, the sigmoid model fitted poorly on somatostatin expression, but an effect of postPP and T_a_ was clearly observable (Figure 6a, Table S2). In spring-programmed voles, somatostatin expression in response to postPP is T_a_ dependent with the higher T_a_ weakened the photoperiodic response (T_a_ × postPP: *F*_3,32_ = 4.703, *p* = 0.008). By contrast, in autumn-programmed voles, photoperiodic effect on somatostatin expression was independent of T_a_. In both groups, long postPPs had an inhibitory effect on somatostatin expression (spring: *F*_3,32_ = 4.56, *p* = 0.009; autumn: *F*_3,28_ = 10.82, *p* < 0.001).

Somatostatin expression across all postPPs did not significantly differ between spring- programmed (0.265 ± 0.004) and autumn-programmed (0.269 ± 0.004) voles or between ambient temperatures 10°C (0.265 ± 0.005) and 21 °C (0.268 ± 0.003) across photoperiod treatments.

### Growth in Response to Somatostatin Expression

Somatostatin expression in the arcuate nucleus was negatively correlated with the postweaning growth potential in both spring-programmed (*F*_1,36_ = 5.31, *p* = 0.03) and autumn-programmed (*F*_1,32_ = 14.51, *p* < 0.001) voles (Figure 6b). Furthermore, low T_a_ (10 °C) in spring led to a significant increase in growth potential despite the growth-suppressive effect of somatostatin (*F*_1,36_ = 7.10, *p* = 0.01).

### Gonadal Activation

**Figure 7.**
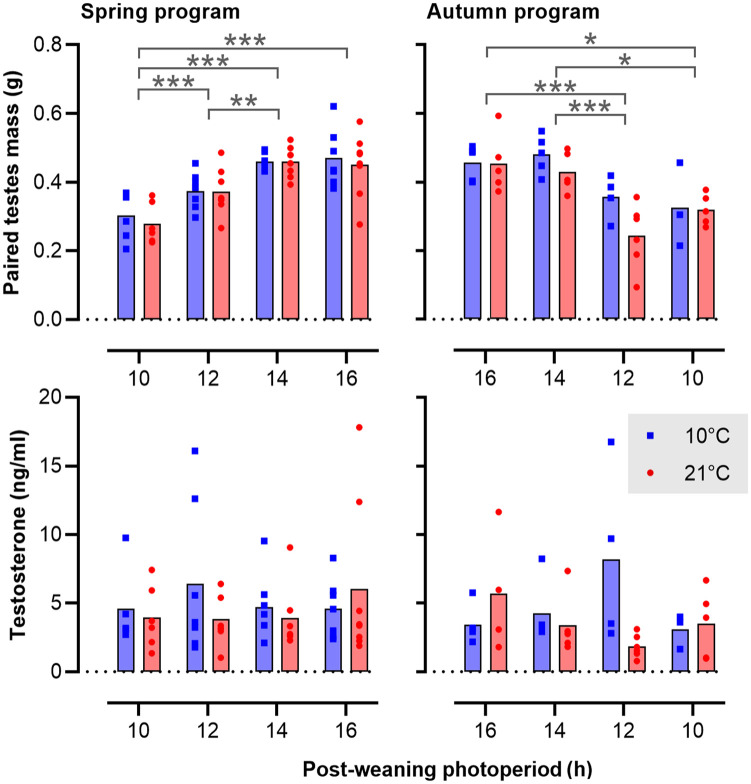
Activation of the gonadal axis in relation to photoperiod and ambient temperature. The bar plots show the mean and values for individual voles. The left panels represent spring-programmed voles (gestated and raised to weaning under 8L) and the right panels represent autumn-programmed voles (gestated and raised to weaning under 16L) at 10 °C (blue squares) or 21 °C (red circles). Abbreviation: ANOVA = analysis of variance. Data were log-transformed prior to analysis by two-way ANOVA. Significant effects are indicated: *p < 0.05. **p < 0.01. ***p < 0.001.

The effects of photoperiod and T_a_ on gonadal weight and end-point plasma testosterone levels are summarized in Figure 7. Independent of gestational photoperiod and T_a_, final gonadal weights in voles kept on a postPP of LD16:8 were consistently about 50% higher than the corresponding values in LD10:14 voles (spring, postPP: *F*_3,51_ = 22.37, *p* < 0.001; autumn, postPP: *F*_3,30_ = 12.18, *p* < 0.001). Contrastingly, no significant effects of T_a_ on testicular growth were observed. Across the study as a whole, wide interindividual variation in testosterone levels at time of sacrifice was observed, and no significant effects of preweaning or postweaning conditions were found.

## Discussion

While there is a considerable literature on the programming effects of photoperiod on postnatal reproductive development in seasonal rodent species (Sáenz de Miera, 2019; van Dalum et al., 2020), few studies have focused on maternal photoperiodic programming of somatic growth (Horton, 2005). Here, we have presented an analysis on this phenomenon in a microtine rodent in which effects on growth are at least as pronounced as those on reproductive development. Our data demonstrate that while gestational photoperiod does not affect body mass prior to weaning, it has a profound effect on postweaning growth parameters. Spring-programmed animals have a higher growth potential (i.e., the theoretical maximum size which an individual will attain) and a longer half-time (i.e., the time taken to attain 50% of the projected maximum weight) than do autumn-programmed animals. Since all animals had ad libitum access to food and water, and the *highest* body masses and growth potentials were seen in voles raised after weaning at a *low* ambient temperature (T_a_), when thermoregulatory energy demands have been higher, we see photoperiodic history-dependent adoption of an autumn growth trajectory as a *programmed* curtailment of the default summer growth trajectory. We suggest that this programming effect facilitates the redirection of dwindling autumn energy supplies toward overwintering survival and is the underlying cause of the numerous field observations of reduced body size linked to fitness in autumn born, overwintering cohorts of voles (Aars and Ims, 2002; Gliwicz, 1996; Zub et al., 2014). Indeed, the temperature effect on body mass disappeared when food was restricted and animals were not able to compensate high energy demands at low T_a_ by increasing food consumption (van Rosmalen and Hut, 2021a).

Low T_a_ enhanced growth potential to a greater extent in spring-programmed voles than in autumn-programmed voles. This asymmetry in the modulatory effect of T_a_ echoes field data on plasticity in the timing of pelage molt in snowshoe hares—where significant weather-dependent plasticity was seen in the completion of the spring but not the autumn molt (Zimova et al., 2014). From a functional perspective, increased programmed rigidity in autumn transitions might reflect high fitness costs of delaying preparation for winter based on unpredictable, and poorly predictive, benign autumn weather conditions. We are unclear as to why low T_a_ should *increase* growth potential in spring-programmed animals. Possibly low T_a_ in the spring condition signals that environmental conditions suitable for reproduction are likely to occur further into the future than in a warm spring, and this in turn encourages a commitment to investing in a large mature body size for later competitiveness. Against this hypothesis, and in contrast to our earlier observations in *M. arvalis* (van Rosmalen et al., 2020, 2021), we observed no corresponding disinvestment in the gonadal axis in low T_a_ spring animals; indeed, development of the gonadal axis in *M. oeconomus* appears to be entirely T_a_ insensitive and only mildly sensitive to photoperiodic influences (see also van Rosmalen et al., 2020, 2021). We have no data on core body temperature (T_b_) or body composition (e.g., fat content) in this study but our previous study showed that T_b_ was lower at a T_a_ of 10 °C compared with 21 °C (van Rosmalen and Hut, 2021b) and another study showed that T_b_ correlates with T_a_ in tundra voles during the winter (Nieminen et al., 2013). The proportion of fat and de novo lipogenesis was not affected by photoperiod-induced body mass changes in *Microtus agrestis* (Król et al., 2005), *Lasiopodomys brandtii* (Li and Wang, 2005), and *M. oeconomus* (Wang et al., 2006), and an increased energy metabolism in winter suggests reliance on continuous food availability rather than fat accumulation in voles (Mustonen et al., 2002). Tundra voles may adapt behaviorally to cope with low temperatures through huddling in tunnels dug under the snow (Korslund, 2006).

Analysis of *dio2*/*dio3* gene expression provides support for the notion that T_a_, gestational and postweaning photoperiodic influences converge at this level. In general terms, this view is consistent with the concept that the tanycyte cells in which the deiodinase genes are expressed are metabolic interfaces to the hypothalamic control systems (Bolborea and Dale, 2013; Dardente et al., 2014). In this study, the observed patterns of *dio2* and *dio3* were broadly consistent with those in the large body of published work describing the effects of seasonal status in the expression of these genes, with the spring condition being *dio2* dominant (*dio2* elevated, *dio3* suppressed) and vice versa in the autumn condition (Dardente et al., 2014; Hazlerigg and Simonneaux, 2015).

It is interesting to note that we observed T_a_ modulation of the expression of *dio2* but not *dio3*, and we therefore speculate that this may be the underlying cause of the asymmetry in the effect of T_a_ on growth potential: T_a_ modulates the *dio2*-dominant spring-programmed state, but not the *dio3*-dominant autumn-programmed state.

In Siberian hamsters, somatostatin (*srif*) expression in the arcuate nucleus shows inverse regulation to the annual body mass cycle (Petri et al., 2016) and treatment with the somatostatin agonist pasireotide mimics the effects of SP exposure on body mass and organ mass (Dumbell et al., 2015). We therefore wondered whether the observed effects of photoperiodic programming on growth parameters might be reflected in effects on *srif* gene expression. While we observed a weak negative correlation between arcuate *srif* expression and growth potential across all treatment groups (Figure 6b), the slope relationship did not change significantly as a function of photoperiodic treatment, and paradoxically we observed increased mean levels of *srif* expression in spring-programmed animals raised at low T_a_, which had the highest overall growth potential. Since our sampling paradigm necessarily limited analysis of brain gene expression to the end of the study, we cannot exclude the possibility of *srif* involvement in growth programming in *M. oeconomus*.

Current models suggest that the regulation of *tshβ* gene expression in the PT represents the key photoperiodic switch for control of seasonal responses (Dardente et al., 2010; Masumoto et al., 2010), and is resistant to photoperiod-independent perturbatory effects—a resistance that might contribute to the function of the PT as a circannual calendar tissue (Lincoln et al., 2003; Wood and Loudon, 2018). In this light, we were surprised to observe a decrease in CP for suppression of *tshβ* expression in autumn-programmed animals held at low T_a_. Given that neither somatic growth potential nor gonadal weights show a similar pattern of response in autumn-programmed animals, it is difficult to put this finding in a functional context. Nevertheless, it raises the possibility that regulation of *tshβ* expression in the PT is more sensitive to metabolic influence than previously appreciated. Moreover, a previous study showed reduced *tshβ* expression in the PT of spring-programmed voles when food is scarce (van Rosmalen and Hut, 2021a).

**Figure 8.**
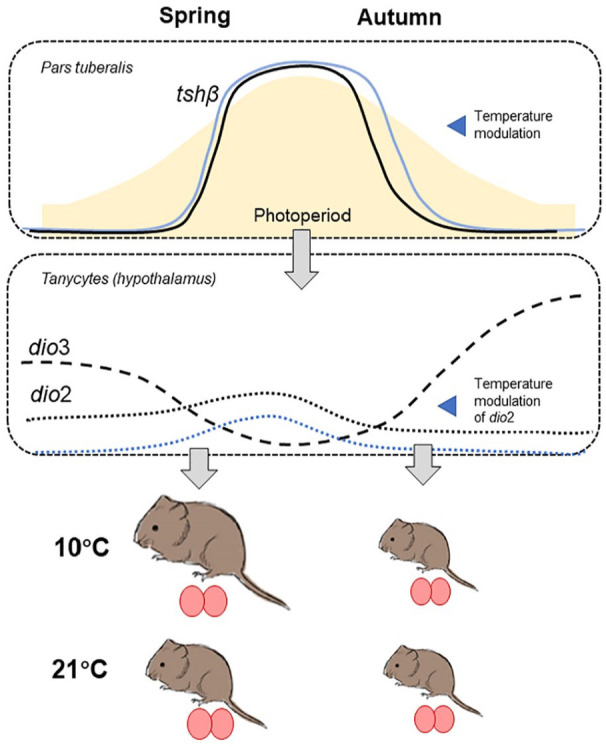
Summary of the photoperiod and temperature effects in tundra voles. *Tshβ* mRNA expression in the *pars tuberalis* follows photoperiod and shows a shift to a shorter (and thus later) critical photoperiod in autumn-programmed voles kept at 10 °C. Asymmetry in postweaning expression between spring and autumn programmed with only *dio2* responding to temperature in spring. This translated to increased growth potential in spring under 10 °C. Ovals indicate testes size which is insensitive to temperature changes and mildly responsive to postweaning photoperiod.

In summary (Figure 8), this study characterizes the programming effect of early life photoperiod on somatic growth in tundra voles and shows that the plasticity of programming in the face of ambient temperature variation is greater in voles following a spring-type growth program. In the brain, this reflected in the temperature dependence of *dio2* gene expression in the mediobasal hypothalamus, and possibly *tshβ* expression in the PT, supporting the concept that this brain area is the key site for integration of metabolic status and calendar time. On a wider scope, our study provides new insights into temperature effects on the growth and reproductive axes of a high-latitude small herbivore. This may aid understanding the adaptive potential of small mammals in the face of climate change and the rising temperatures at higher latitudes.

## Research Data

sj-csv-1-jbr-10.1177_07487304231190156 – for Ambient Temperature Effects on the Spring and Autumn Somatic Growth Trajectory Show Plasticity in the Photoneuroendocrine Response Pathway in the Tundra Vole Click here for additional data file. sj-csv-1-jbr-10.1177_07487304231190156 for Ambient Temperature Effects on the Spring and Autumn Somatic Growth Trajectory Show Plasticity in the Photoneuroendocrine Response Pathway in the Tundra Vole by Mattis Jayme van Dalum, Laura van Rosmalen, Daniel Appenroth, Fernando Cazarez Marquez, Renzo T. M. Roodenrijs, Lauren de Wit, Roelof A. Hut and David G. Hazlerigg in Journal of Biological Rhythms This article is distributed under the terms of the Creative Commons Attribution 4.0 License ( http://www.creativecommons.org/licenses/by/4.0/) which permits any use, reproduction and distribution of the work without further permission provided the original work is attributed as specified on the SAGE and Open Access pages ( https://us.sagepub.com/en-us/nam/open-access-at-sage).

sj-docx-2-jbr-10.1177_07487304231190156 – Supplemental material for Ambient Temperature Effects on the Spring and Autumn Somatic Growth Trajectory Show Plasticity in the Photoneuroendocrine Response Pathway in the Tundra Vole Click here for additional data file. Supplemental material, sj-docx-2-jbr-10.1177_07487304231190156 for Ambient Temperature Effects on the Spring and Autumn Somatic Growth Trajectory Show Plasticity in the Photoneuroendocrine Response Pathway in the Tundra Vole by Mattis Jayme van Dalum, Laura van Rosmalen, Daniel Appenroth, Fernando Cazarez Marquez, Renzo T. M. Roodenrijs, Lauren de Wit, Roelof A. Hut and David G. Hazlerigg in Journal of Biological Rhythms
