# Supplementary material for: Ambient Temperature Effects on the Spring and Autumn Somatic Growth Trajectory Show Plasticity in the Photoneuroendocrine Response Pathway in the Tundra Vole
Source: J Biol Rhythms. 2023 Aug 11;38(6):586–600. doi: 10.1177/07487304231190156 (PMC10617003; doi:10.1177/07487304231190156)
Supplement: sj-docx-2-jbr-10.1177_07487304231190156 – Supplemental material for Ambient Temperature Effects on the Spring and Autumn Somatic Growth Trajectory Show Plasticity in the Photoneuroendocrine Response Pathway in the Tundra Vole [file sj-docx-2-jbr-10.1177_07487304231190156.docx]

**Table S1: descriptive statistics for all variables**

| variable | Prew PP(h) | T_a_ ( ̊C) | Postw PP (h) | sample size | mean | sd | 95% CI lower | 95% CI upper | min | Q1 | median | Q3 | max |
| --- | --- | --- | --- | --- | --- | --- | --- | --- | --- | --- | --- | --- | --- |
| p50 body mass (g) | 8 | 10 | 10 | 6 | 29.92 | 2.64 | 27.14 | 32.69 | 25.95 | 28.11 | 30.98 | 31.88 | 32.26 |
|  | 8 | 10 | 12 | 9 | 31.42 | 2.66 | 29.38 | 33.46 | 26.74 | 29.89 | 31.85 | 32.18 | 36.53 |
|  | 8 | 10 | 14 | 7 | 35.82 | 4.09 | 32.04 | 39.60 | 31.12 | 32.42 | 35.92 | 38.44 | 42.01 |
|  | 8 | 10 | 16 | 7 | 37.56 | 6.12 | 31.90 | 43.23 | 28.66 | 33.21 | 37.96 | 42.56 | 44.79 |
|  | 8 | 21 | 10 | 7 | 27.22 | 4.10 | 23.43 | 31.01 | 23.06 | 24.11 | 25.01 | 30.38 | 33.48 |
|  | 8 | 21 | 12 | 8 | 30.45 | 4.11 | 27.01 | 33.89 | 24.03 | 27.63 | 30.73 | 32.53 | 36.77 |
|  | 8 | 21 | 14 | 8 | 37.89 | 5.32 | 33.44 | 42.33 | 33.52 | 34.12 | 35.94 | 39.56 | 48.75 |
|  | 8 | 21 | 16 | 8 | 34.37 | 6.42 | 29.00 | 39.74 | 26.60 | 29.41 | 34.45 | 37.98 | 45.64 |
|  | 16 | 10 | 10 | 3 | 29.01 | 10.07 | 3.98 | 54.04 | 22.26 | 23.22 | 24.18 | 32.39 | 40.59 |
|  | 16 | 10 | 12 | 4 | 30.64 | 4.71 | 23.14 | 38.14 | 26.16 | 27.32 | 29.88 | 33.19 | 36.64 |
|  | 16 | 10 | 14 | 5 | 34.44 | 4.48 | 28.89 | 40.00 | 30.39 | 31.61 | 32.38 | 36.54 | 41.30 |
|  | 16 | 10 | 16 | 5 | 33.81 | 4.92 | 27.70 | 39.92 | 28.40 | 29.18 | 34.32 | 38.17 | 38.99 |
|  | 16 | 21 | 10 | 5 | 27.94 | 3.25 | 23.91 | 31.97 | 23.26 | 27.05 | 28.01 | 29.22 | 32.16 |
|  | 16 | 21 | 12 | 6 | 23.29 | 4.19 | 18.89 | 27.68 | 18.41 | 20.07 | 23.51 | 25.15 | 29.68 |
|  | 16 | 21 | 14 | 5 | 34.05 | 2.46 | 31.00 | 37.10 | 30.72 | 33.27 | 33.33 | 36.06 | 36.87 |
|  | 16 | 21 | 16 | 9 | 34.50 | 4.31 | 31.19 | 37.81 | 28.24 | 31.98 | 33.63 | 35.76 | 43.38 |
| growth potential (g) | 8 | 10 | 10 | 6 | 18.52 | 5.97 | 12.26 | 24.79 | 10.56 | 14.36 | 18.57 | 23.59 | 25.23 |
|  | 8 | 10 | 12 | 9 | 23.85 | 8.53 | 17.29 | 30.41 | 15.49 | 20.69 | 22.46 | 23.15 | 45.51 |
|  | 8 | 10 | 14 | 7 | 27.19 | 7.23 | 20.51 | 33.88 | 13.05 | 25.06 | 29.64 | 31.22 | 35.11 |
|  | 8 | 10 | 16 | 7 | 32.39 | 14.23 | 19.23 | 45.55 | 17.30 | 23.25 | 29.02 | 38.07 | 57.75 |
|  | 8 | 21 | 10 | 7 | 12.56 | 5.03 | 7.91 | 17.22 | 6.72 | 9.17 | 11.07 | 15.64 | 20.52 |
|  | 8 | 21 | 12 | 8 | 16.07 | 5.70 | 11.30 | 20.84 | 10.06 | 12.06 | 14.67 | 18.55 | 26.61 |
|  | 8 | 21 | 14 | 8 | 25.41 | 11.03 | 16.19 | 34.63 | 14.94 | 17.62 | 22.33 | 29.37 | 47.46 |
|  | 8 | 21 | 16 | 8 | 21.13 | 6.84 | 15.41 | 26.84 | 11.32 | 15.64 | 21.36 | 27.34 | 29.82 |
|  | 16 | 10 | 10 | 3 | 10.33 | 10.09 | -14.74 | 35.40 | 3.43 | 4.54 | 5.66 | 13.78 | 21.91 |
|  | 16 | 10 | 12 | 4 | 14.39 | 4.98 | 6.47 | 22.31 | 9.50 | 11.97 | 13.38 | 15.81 | 21.30 |
|  | 16 | 10 | 14 | 5 | 16.53 | 2.47 | 13.46 | 19.60 | 12.62 | 15.64 | 17.50 | 18.44 | 18.47 |
|  | 16 | 10 | 16 | 5 | 19.54 | 3.60 | 15.07 | 24.02 | 15.68 | 18.04 | 18.06 | 20.85 | 25.09 |
|  | 16 | 21 | 10 | 5 | 10.77 | 2.15 | 8.10 | 13.44 | 7.39 | 10.42 | 10.79 | 12.47 | 12.78 |
|  | 16 | 21 | 12 | 6 | 8.83 | 2.66 | 6.04 | 11.61 | 6.50 | 6.72 | 8.19 | 10.13 | 13.09 |
|  | 16 | 21 | 14 | 5 | 16.14 | 2.68 | 12.82 | 19.47 | 12.61 | 14.48 | 16.18 | 18.47 | 18.97 |
|  | 16 | 21 | 16 | 8 | 19.27 | 7.68 | 12.84 | 25.69 | 11.30 | 13.86 | 17.27 | 23.31 | 34.34 |
| growth rate (half time) | 8 | 10 | 10 | 6 | 13.15 | 6.52 | 6.30 | 19.99 | 6.94 | 8.18 | 12.20 | 15.29 | 24.38 |
|  | 8 | 10 | 12 | 9 | 15.25 | 11.97 | 6.05 | 24.45 | 5.63 | 9.40 | 12.06 | 14.85 | 45.91 |
|  | 8 | 10 | 14 | 7 | 12.90 | 7.11 | 6.32 | 19.48 | 2.64 | 9.32 | 13.05 | 15.68 | 24.61 |
|  | 8 | 10 | 16 | 7 | 15.16 | 9.51 | 6.37 | 23.95 | 6.69 | 8.11 | 9.57 | 21.30 | 31.06 |
|  | 8 | 21 | 10 | 7 | 11.07 | 5.86 | 5.65 | 16.49 | 4.10 | 6.48 | 10.93 | 15.63 | 18.23 |
|  | 8 | 21 | 12 | 8 | 9.32 | 5.00 | 5.14 | 13.50 | 3.37 | 5.92 | 8.81 | 11.86 | 18.65 |
|  | 8 | 21 | 14 | 8 | 13.95 | 14.37 | 1.94 | 25.96 | 3.92 | 6.91 | 9.95 | 12.35 | 48.61 |
|  | 8 | 21 | 16 | 8 | 8.68 | 6.38 | 3.35 | 14.02 | 0.00 | 5.50 | 7.12 | 12.22 | 20.61 |
|  | 16 | 10 | 10 | 3 | 2.41 | 1.82 | -2.11 | 6.94 | 0.96 | 1.39 | 1.83 | 3.14 | 4.45 |
|  | 16 | 10 | 12 | 4 | 4.19 | 3.39 | -1.21 | 9.58 | 0.00 | 2.27 | 4.53 | 6.45 | 7.68 |
|  | 16 | 10 | 14 | 5 | 4.51 | 0.98 | 3.29 | 5.72 | 3.63 | 3.86 | 4.45 | 4.46 | 6.14 |
|  | 16 | 10 | 16 | 5 | 6.76 | 2.87 | 3.20 | 10.32 | 4.39 | 4.43 | 5.33 | 9.12 | 10.53 |
|  | 16 | 21 | 10 | 5 | 2.57 | 2.86 | -0.99 | 6.13 | 0.00 | 0.00 | 2.52 | 3.42 | 6.91 |
|  | 16 | 21 | 12 | 6 | 4.96 | 3.40 | 1.39 | 8.53 | 2.22 | 2.70 | 3.79 | 5.77 | 11.18 |
|  | 16 | 21 | 14 | 5 | 3.95 | 2.42 | 0.95 | 6.96 | 0.00 | 3.77 | 4.26 | 5.51 | 6.24 |
|  | 16 | 21 | 16 | 8 | 8.00 | 6.41 | 2.64 | 13.36 | 0.00 | 4.50 | 6.79 | 9.52 | 21.48 |
| Somatostatin expression (OD) | 8 | 10 | 10 | 4 | 0.30 | 0.02 | 0.27 | 0.34 | 0.27 | 0.30 | 0.31 | 0.32 | 0.32 |
|  | 8 | 10 | 12 | 4 | 0.27 | 0.03 | 0.23 | 0.32 | 0.24 | 0.26 | 0.27 | 0.29 | 0.31 |
|  | 8 | 10 | 14 | 3 | 0.24 | 0.02 | 0.20 | 0.28 | 0.23 | 0.23 | 0.24 | 0.25 | 0.26 |
|  | 8 | 10 | 16 | 4 | 0.24 | 0.01 | 0.23 | 0.25 | 0.24 | 0.24 | 0.24 | 0.24 | 0.25 |
|  | 8 | 21 | 10 | 6 | 0.27 | 0.03 | 0.24 | 0.30 | 0.24 | 0.25 | 0.28 | 0.29 | 0.31 |
|  | 8 | 21 | 12 | 7 | 0.26 | 0.01 | 0.25 | 0.27 | 0.25 | 0.25 | 0.26 | 0.26 | 0.29 |
|  | 8 | 21 | 14 | 7 | 0.26 | 0.01 | 0.25 | 0.28 | 0.24 | 0.25 | 0.27 | 0.27 | 0.28 |
|  | 8 | 21 | 16 | 6 | 0.27 | 0.02 | 0.25 | 0.28 | 0.24 | 0.26 | 0.27 | 0.27 | 0.29 |
|  | 16 | 10 | 10 | 3 | 0.30 | 0.03 | 0.21 | 0.38 | 0.26 | 0.28 | 0.30 | 0.31 | 0.33 |
|  | 16 | 10 | 12 | 4 | 0.27 | 0.01 | 0.25 | 0.29 | 0.26 | 0.27 | 0.27 | 0.28 | 0.29 |
|  | 16 | 10 | 14 | 5 | 0.26 | 0.01 | 0.25 | 0.28 | 0.25 | 0.25 | 0.26 | 0.26 | 0.28 |
|  | 16 | 10 | 16 | 4 | 0.24 | 0.02 | 0.20 | 0.27 | 0.21 | 0.22 | 0.24 | 0.25 | 0.26 |
|  | 16 | 21 | 10 | 5 | 0.29 | 0.02 | 0.27 | 0.31 | 0.27 | 0.28 | 0.28 | 0.30 | 0.31 |
|  | 16 | 21 | 12 | 6 | 0.29 | 0.02 | 0.26 | 0.31 | 0.25 | 0.28 | 0.29 | 0.30 | 0.31 |
|  | 16 | 21 | 14 | 4 | 0.27 | 0.02 | 0.23 | 0.30 | 0.24 | 0.26 | 0.27 | 0.27 | 0.29 |
|  | 16 | 21 | 16 | 5 | 0.25 | 0.01 | 0.24 | 0.26 | 0.23 | 0.25 | 0.25 | 0.26 | 0.26 |
| tshβ expression (OD) | 8 | 10 | 10 | 4 | 0.02 | 0.03 | -0.03 | 0.06 | 0.00 | 0.00 | 0.01 | 0.02 | 0.06 |
|  | 8 | 10 | 12 | 4 | 0.04 | 0.07 | -0.07 | 0.16 | 0.00 | 0.01 | 0.01 | 0.05 | 0.15 |
|  | 8 | 10 | 14 | 4 | 0.33 | 0.19 | 0.02 | 0.64 | 0.05 | 0.30 | 0.39 | 0.42 | 0.49 |
|  | 8 | 10 | 16 | 4 | 0.59 | 0.21 | 0.26 | 0.93 | 0.28 | 0.57 | 0.69 | 0.72 | 0.72 |
|  | 8 | 21 | 10 | 6 | 0.00 | 0.01 | 0.00 | 0.01 | 0.00 | 0.00 | 0.00 | 0.01 | 0.01 |
|  | 8 | 21 | 12 | 7 | 0.01 | 0.01 | 0.00 | 0.01 | 0.00 | 0.00 | 0.01 | 0.01 | 0.02 |
|  | 8 | 21 | 14 | 7 | 0.17 | 0.14 | 0.04 | 0.30 | 0.00 | 0.05 | 0.19 | 0.29 | 0.33 |
|  | 8 | 21 | 16 | 7 | 0.60 | 0.12 | 0.49 | 0.71 | 0.37 | 0.57 | 0.59 | 0.67 | 0.73 |
|  | 16 | 10 | 10 | 3 | 0.00 | 0.00 | 0.00 | 0.00 | 0.00 | 0.00 | 0.00 | 0.00 | 0.00 |
|  | 16 | 10 | 12 | 4 | 0.02 | 0.02 | -0.01 | 0.05 | 0.01 | 0.01 | 0.01 | 0.02 | 0.04 |
|  | 16 | 10 | 14 | 5 | 0.45 | 0.23 | 0.16 | 0.74 | 0.15 | 0.39 | 0.41 | 0.52 | 0.79 |
|  | 16 | 10 | 16 | 5 | 0.73 | 0.05 | 0.67 | 0.79 | 0.66 | 0.73 | 0.74 | 0.76 | 0.79 |
|  | 16 | 21 | 10 | 5 | 0.01 | 0.01 | -0.01 | 0.02 | 0.00 | 0.00 | 0.00 | 0.01 | 0.02 |
|  | 16 | 21 | 12 | 6 | 0.00 | 0.00 | 0.00 | 0.01 | 0.00 | 0.00 | 0.00 | 0.01 | 0.01 |
|  | 16 | 21 | 14 | 4 | 0.16 | 0.04 | 0.09 | 0.22 | 0.11 | 0.13 | 0.16 | 0.18 | 0.21 |
|  | 16 | 21 | 16 | 5 | 0.56 | 0.13 | 0.40 | 0.72 | 0.37 | 0.51 | 0.57 | 0.61 | 0.72 |
| dio2 expression (OD) | 8 | 10 | 10 | 4 | 0.00 | 0.00 | 0.00 | 0.00 | 0.00 | 0.00 | 0.00 | 0.00 | 0.00 |
|  | 8 | 10 | 12 | 4 | 0.00 | 0.00 | 0.00 | 0.00 | 0.00 | 0.00 | 0.00 | 0.00 | 0.00 |
|  | 8 | 10 | 14 | 4 | 0.07 | 0.13 | -0.13 | 0.28 | 0.00 | 0.00 | 0.01 | 0.08 | 0.27 |
|  | 8 | 10 | 16 | 4 | 0.05 | 0.05 | -0.04 | 0.13 | 0.01 | 0.01 | 0.04 | 0.08 | 0.11 |
|  | 8 | 21 | 10 | 7 | 0.06 | 0.06 | 0.00 | 0.11 | 0.01 | 0.02 | 0.03 | 0.08 | 0.17 |
|  | 8 | 21 | 12 | 8 | 0.03 | 0.04 | 0.00 | 0.06 | 0.00 | 0.00 | 0.01 | 0.04 | 0.09 |
|  | 8 | 21 | 14 | 8 | 0.12 | 0.15 | 0.00 | 0.24 | 0.01 | 0.03 | 0.05 | 0.16 | 0.36 |
|  | 8 | 21 | 16 | 8 | 0.12 | 0.09 | 0.05 | 0.20 | 0.01 | 0.07 | 0.12 | 0.16 | 0.28 |
|  | 16 | 10 | 10 | 3 | 0.00 | 0.00 | 0.00 | 0.00 | 0.00 | 0.00 | 0.00 | 0.00 | 0.00 |
|  | 16 | 10 | 12 | 4 | 0.01 | 0.02 | -0.02 | 0.04 | 0.00 | 0.00 | 0.00 | 0.01 | 0.03 |
|  | 16 | 10 | 14 | 5 | 0.00 | 0.00 | 0.00 | 0.00 | 0.00 | 0.00 | 0.00 | 0.00 | 0.00 |
|  | 16 | 10 | 16 | 5 | 0.00 | 0.01 | 0.00 | 0.01 | 0.00 | 0.00 | 0.00 | 0.00 | 0.02 |
|  | 16 | 21 | 10 | 5 | 0.02 | 0.02 | 0.00 | 0.04 | 0.01 | 0.01 | 0.01 | 0.04 | 0.04 |
|  | 16 | 21 | 12 | 6 | 0.04 | 0.03 | 0.01 | 0.08 | 0.00 | 0.03 | 0.04 | 0.05 | 0.10 |
|  | 16 | 21 | 14 | 4 | 0.05 | 0.01 | 0.03 | 0.07 | 0.04 | 0.04 | 0.05 | 0.05 | 0.06 |
|  | 16 | 21 | 16 | 5 | 0.03 | 0.02 | 0.01 | 0.05 | 0.01 | 0.02 | 0.03 | 0.04 | 0.06 |
| dio3 expression (OD) | 8 | 10 | 10 | 4 | 0.09 | 0.05 | 0.01 | 0.17 | 0.04 | 0.05 | 0.09 | 0.13 | 0.14 |
|  | 8 | 10 | 12 | 4 | 0.02 | 0.01 | 0.00 | 0.04 | 0.01 | 0.01 | 0.02 | 0.03 | 0.03 |
|  | 8 | 10 | 14 | 4 | 0.02 | 0.01 | 0.00 | 0.04 | 0.00 | 0.01 | 0.02 | 0.03 | 0.03 |
|  | 8 | 10 | 16 | 4 | 0.02 | 0.01 | 0.00 | 0.04 | 0.00 | 0.01 | 0.02 | 0.02 | 0.04 |
|  | 8 | 21 | 10 | 7 | 0.19 | 0.12 | 0.08 | 0.31 | 0.00 | 0.13 | 0.20 | 0.27 | 0.36 |
|  | 8 | 21 | 12 | 8 | 0.08 | 0.08 | 0.01 | 0.14 | 0.00 | 0.01 | 0.05 | 0.13 | 0.22 |
|  | 8 | 21 | 14 | 8 | 0.04 | 0.03 | 0.01 | 0.06 | 0.00 | 0.01 | 0.03 | 0.06 | 0.07 |
|  | 8 | 21 | 16 | 7 | 0.02 | 0.02 | 0.00 | 0.04 | 0.00 | 0.01 | 0.01 | 0.02 | 0.06 |
|  | 16 | 10 | 10 | 3 | 0.45 | 0.10 | 0.20 | 0.70 | 0.34 | 0.41 | 0.48 | 0.51 | 0.53 |
|  | 16 | 10 | 12 | 4 | 0.40 | 0.21 | 0.07 | 0.73 | 0.19 | 0.24 | 0.40 | 0.56 | 0.61 |
|  | 16 | 10 | 14 | 5 | 0.13 | 0.21 | -0.13 | 0.39 | 0.00 | 0.00 | 0.07 | 0.09 | 0.50 |
|  | 16 | 10 | 16 | 5 | 0.03 | 0.03 | 0.00 | 0.06 | 0.00 | 0.01 | 0.04 | 0.05 | 0.05 |
|  | 16 | 21 | 10 | 5 | 0.45 | 0.25 | 0.14 | 0.77 | 0.16 | 0.30 | 0.38 | 0.63 | 0.79 |
|  | 16 | 21 | 12 | 6 | 0.40 | 0.12 | 0.27 | 0.53 | 0.27 | 0.30 | 0.39 | 0.45 | 0.60 |
|  | 16 | 21 | 14 | 4 | 0.11 | 0.04 | 0.05 | 0.16 | 0.07 | 0.08 | 0.10 | 0.13 | 0.14 |
|  | 16 | 21 | 16 | 5 | 0.07 | 0.10 | -0.04 | 0.19 | 0.00 | 0.00 | 0.04 | 0.10 | 0.23 |
| paired testes mass (g) | 8 | 10 | 10 | 6 | 0.30 | 0.07 | 0.23 | 0.38 | 0.21 | 0.26 | 0.32 | 0.36 | 0.37 |
|  | 8 | 10 | 12 | 9 | 0.37 | 0.05 | 0.34 | 0.41 | 0.30 | 0.35 | 0.38 | 0.40 | 0.45 |
|  | 8 | 10 | 14 | 7 | 0.46 | 0.02 | 0.44 | 0.48 | 0.43 | 0.44 | 0.46 | 0.48 | 0.49 |
|  | 8 | 10 | 16 | 7 | 0.47 | 0.08 | 0.39 | 0.55 | 0.38 | 0.42 | 0.44 | 0.51 | 0.62 |
|  | 8 | 21 | 10 | 7 | 0.28 | 0.05 | 0.23 | 0.33 | 0.23 | 0.24 | 0.26 | 0.32 | 0.36 |
|  | 8 | 21 | 12 | 8 | 0.37 | 0.07 | 0.32 | 0.43 | 0.27 | 0.34 | 0.35 | 0.41 | 0.49 |
|  | 8 | 21 | 14 | 8 | 0.46 | 0.04 | 0.42 | 0.50 | 0.39 | 0.43 | 0.47 | 0.48 | 0.52 |
|  | 8 | 21 | 16 | 8 | 0.45 | 0.09 | 0.37 | 0.53 | 0.28 | 0.43 | 0.47 | 0.49 | 0.58 |
|  | 16 | 10 | 10 | 3 | 0.33 | 0.12 | 0.02 | 0.63 | 0.21 | 0.26 | 0.31 | 0.38 | 0.46 |
|  | 16 | 10 | 12 | 4 | 0.36 | 0.06 | 0.26 | 0.46 | 0.27 | 0.33 | 0.37 | 0.39 | 0.42 |
|  | 16 | 10 | 14 | 5 | 0.48 | 0.06 | 0.41 | 0.55 | 0.41 | 0.45 | 0.49 | 0.52 | 0.55 |
|  | 16 | 10 | 16 | 5 | 0.46 | 0.05 | 0.39 | 0.52 | 0.40 | 0.40 | 0.49 | 0.49 | 0.50 |
|  | 16 | 21 | 10 | 5 | 0.32 | 0.05 | 0.26 | 0.38 | 0.27 | 0.29 | 0.32 | 0.35 | 0.38 |
|  | 16 | 21 | 12 | 6 | 0.24 | 0.09 | 0.15 | 0.34 | 0.09 | 0.20 | 0.26 | 0.30 | 0.36 |
|  | 16 | 21 | 14 | 5 | 0.43 | 0.06 | 0.36 | 0.50 | 0.36 | 0.40 | 0.41 | 0.48 | 0.50 |
|  | 16 | 21 | 16 | 9 | 0.44 | 0.06 | 0.39 | 0.49 | 0.37 | 0.40 | 0.43 | 0.45 | 0.59 |
| testosterone (ng/ml) | 8 | 10 | 10 | 5 | 4.59 | 2.94 | 0.94 | 8.24 | 2.70 | 3.10 | 3.17 | 4.22 | 9.75 |
|  | 8 | 10 | 12 | 7 | 6.42 | 5.64 | 1.20 | 11.64 | 1.77 | 2.65 | 3.61 | 9.09 | 16.10 |
|  | 8 | 10 | 14 | 7 | 4.72 | 2.41 | 2.49 | 6.94 | 2.10 | 3.36 | 4.20 | 5.24 | 9.52 |
|  | 8 | 10 | 16 | 7 | 4.60 | 2.16 | 2.60 | 6.60 | 2.38 | 2.76 | 4.56 | 5.73 | 8.28 |
|  | 8 | 21 | 10 | 6 | 3.96 | 2.30 | 1.55 | 6.38 | 1.36 | 2.42 | 3.46 | 5.37 | 7.42 |
|  | 8 | 21 | 12 | 8 | 3.84 | 1.75 | 2.38 | 5.30 | 1.04 | 3.04 | 3.25 | 5.40 | 6.39 |
|  | 8 | 21 | 14 | 8 | 3.91 | 2.26 | 2.03 | 5.80 | 2.28 | 2.53 | 3.03 | 4.44 | 9.06 |
|  | 8 | 21 | 16 | 8 | 6.02 | 5.84 | 1.13 | 10.90 | 1.90 | 2.47 | 3.41 | 6.42 | 17.81 |
|  | 16 | 10 | 10 | 3 | 3.09 | 1.26 | -0.04 | 6.22 | 1.65 | 2.63 | 3.61 | 3.81 | 4.01 |
|  | 16 | 10 | 12 | 4 | 8.19 | 6.49 | -2.13 | 18.52 | 2.80 | 3.34 | 6.61 | 11.46 | 16.74 |
|  | 16 | 10 | 14 | 5 | 4.27 | 2.22 | 1.51 | 7.03 | 2.90 | 3.33 | 3.42 | 3.46 | 8.22 |
|  | 16 | 10 | 16 | 5 | 3.43 | 1.36 | 1.74 | 5.12 | 2.18 | 2.90 | 3.09 | 3.20 | 5.75 |
|  | 16 | 21 | 10 | 5 | 3.51 | 2.49 | 0.42 | 6.61 | 0.98 | 1.03 | 3.94 | 4.95 | 6.67 |
|  | 16 | 21 | 12 | 6 | 1.85 | 0.85 | 0.96 | 2.73 | 0.78 | 1.37 | 1.67 | 2.35 | 3.11 |
|  | 16 | 21 | 14 | 5 | 3.40 | 2.25 | 0.60 | 6.19 | 1.82 | 2.10 | 2.76 | 2.95 | 7.34 |
|  | 16 | 21 | 16 | 5 | 5.69 | 3.80 | 0.98 | 10.40 | 1.79 | 3.08 | 5.96 | 5.96 | 11.65 |

**Table S2: curve fits for gene expression**

Curve-fitting statistics were calculated with Graphpad Prism. For *Dio2*, *Dio3* and somatostatin, the curve fit was suboptimal hence the missing values where the model failed to calculate certain parameters or confidence levels. These are indicated with ‘NA‘.

|  |  |  | Hillslope (b) | | Bottom (c) | Top (d) |  | EC50 (e) | |  |
| --- | --- | --- | --- | --- | --- | --- | --- | --- | --- | --- |
| Variable | Prew PP  (h) | T_a_ ( ̊C) |  | 95% CI |  |  | 95% CI |  | 95% CI | R^2^ (df) |
| TSHBb | 8 | 10 | 11,24 | 4,72 to 17,77 | *0* | *0,85* |  | 14,73 | 14,01 to 15,46 | 0,76 (14) |
|  | 16 | 10 | 17,68 | 12,64 to 27,43 | *0* | *0,85* |  | 14,38 | 14,09 to 14,69 | 0,93 (15) |
|  | 8 | 21 | 16,92 | 12,74 to 22,29 | *0* | *0,85* |  | 15,21 | 14,93 to 15,49 | 0,88 (25) |
|  | 16 | 21 | 16,27 | 12,46 to 21,50 | *0* | *0,85* |  | 15,38 | 15,12 to 15,63 | 0,93 (18) |
| Dio2 | 8 | 10 | 5,98 | -4,94 to 31,22 | *0* | *0,12* |  | 15,69 | 11,70 to NA | 0,11(14) |
|  | 16 | 10 | 0,01 | 0,01 to NA | *0* | *0,12* |  | NA | NA | 0,00 (15) |
|  | 8 | 21 | 0,00 | 0,00 to 0,00 | *0* | *0,12* |  | NA | NA | 0,00 (29) |
|  | 16 | 21 | 0,67 | -1,73 to 3,21 | *0* | *0,12* |  | 45,48 | 16,36 to NA | 0,02 (18) |
| Dio3 | 8 | 10 | -6,46 | NA to -2,67 | *0* | *0,5* |  | 7,88 | 4,93 to NA | 0,58 (14) |
|  | 16 | 10 | -14,58 | NA to -5,46 | *0* | *0,5* |  | 13,09 | 11,94 to 14,07 | 0,60 (15) |
|  | 8 | 21 | -6,48 | -11,92 to -3,47 | *0* | *0,5* |  | 9,29 | 7,78 to 9,96 | 0,47 (28) |
|  | 16 | 21 | -13,44 | -46,21 to -5,18 | *0* | *0,5* |  | 13,06 | 12,12 to 14,19 | 0,58 (18) |
| Somatostatin | 8 | 10 | 0,35 | -0.73 to 1,43 | *0* | 0.54 | -1,04 to 2,11 | 2,08 | -32,99 to 37.15 | 0,02 (12) |
|  | 16 | 10 | 1,50 | -1,24 to 4,24 | *0* | 0,30 | 0,24 to 0.37 | 9,41 | -1,40 to 20,23 | 0,02 (13) |
|  | 8 | 21 | 0,023 | NA | *0* | 0,53 | NA | 1,29 | NA | 0,01 (22) |
|  | 16 | 21 | 2,52 | -0,24 to 5,28 | *0* | *0,29* | *0,28 to 0,31* | 8,05 | 2,29 to 13,80 | 0,02 (17 |
